# Impetigo herpetiformis: a rare dermotosis of pregnancy

**DOI:** 10.11604/pamj.2018.30.273.16424

**Published:** 2018-08-10

**Authors:** Amal El Fiboumi, Soumya Chiheb

**Affiliations:** 1 Dermatology Department, UHC Ibn Rochd, University of Medicine Hassan II, Casablanca, Morocco

**Keywords:** Impetigo herpetiformis, dermatosis of pregnancy, pustular dermatosis

## Image in medicine

Impetigo herpetiformis (IH) is a rare pustular dermatosis. It is actually considered as a rare subtype of generalized pustular psoriasis occurring in pregnancy. It can be life threatening for both mother and fetus and often causes therapeutic problems. It is manifested by generalized erythematous plaques lined with sterile pustules with alteration of general state. It must be distinguished from specific dermatosis of pregnancy (polymorphic eruption of pregnancy, pemphigoid gestationis) and the other pustular lesions occurring during acute generalized exanthematous pustulosis and hypocalcemiae or infection. Early diagnosis and treatment is recommended. We report a case of a 30 year-old patient who presented at 27 weeks of her first pregnancy, erythematous macular lesion in the inguinal fold, extending in a week to the rest of the body with fever and asthenia. Physical examination found well-defined erythematous plaques bordered by pustules in the face, limbs and large folds and confluent at the trunk without mucosal involvement. Laboratory test showed sedimentation rate at 30mm, CRP at 50mg/l, leucocytes at 9000e/l and slight hypocalcemia due to a hypoalbunimia. Bacteriological sampling of pus was negative. Histopathology revealed spongiform pustule in the epidermis consistent with IH. Prenatal ultrasound did not show growth retardation. We prescribed oral prednisone at the dose of 0.5mg/kg/day with serious monitoring of the fetus. After 2 weeks she had improved and delivered later a healthy female baby with normal birth weight at 37 weeks of pregnancy.

**Figure 1:**
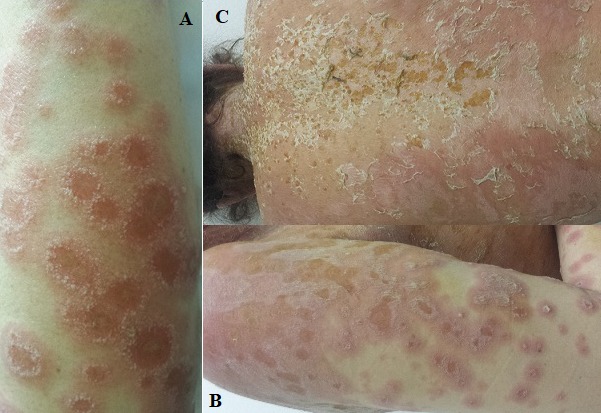
(A,B) erythematous plaques bordered by pustules; (C) confluent erythema with desquamation

